# Optimization and Performance of Sustainable Mortar Incorporating High-Volume Alkali Bypass Dust: A Synergistic Approach Using Silica Fume and Water Reducer

**DOI:** 10.3390/ma19112408

**Published:** 2026-06-05

**Authors:** Riyadh Alturki, Muhammad Imran Khan

**Affiliations:** Civil Engineering Department, College of Engineering, Imam Mohammad Ibn Saud Islamic University (IMSIU), Riyadh 11564, Saudi Arabia; mekhan@imamu.edu.sa

**Keywords:** environmental sustainability, Alkali bypass dust (ABD), sustainable mortar, Response Surface Methodology (RSM), ANOVA, synergistic effect

## Abstract

This study investigates the use of Alkali Bypass Dust (ABD), a cement kiln waste, as a supplementary cementitious material in mortar. Direct ABD incorporation reduced workability and strength. A dual-modification strategy employing a water reducer (WR) and silica fume (SF) was implemented. Mortars with 0–50% cement replaced by ABD were tested, with and without modifiers. Results showed that WR effectively restored workability and improved early strength, while SF enhanced long-term performance through pozzolanic reactions. A synergistic effect in ternary blends (ABD + WR + SF) yielded 28-day compressive strength at 50% ABD replacement comparable to the control (49.9 MPa). Statistical analysis via Response Surface Methodology confirmed that material interactions, not individual amounts, primarily govern strength development. All models are significant where R^2^ value is higher than 0.80. The statistically validated models can be used to optimize the mix proportions for desired compressive and flexural performance. The study concludes that optimized blends with 30–50% ABD are viable for non-structural applications, offering a sustainable pathway for waste valorization and reduced cement consumption.

## 1. Introduction

The cement industry is a major contributor to global anthropogenic CO_2_ emissions, accounting for approximately 7–8% of the total [1]. Cement is produced by processing calcareous materials, such as limestone, and argillaceous materials, like clay or shale, which provide essential oxides for clinker formation [2]. However, these raw materials contain impurities such as potassium (K) and sodium (Na). During the high-temperature clinkerization process, these alkalis volatilize in the kiln’s burning zone [3] and subsequently condense on cooler feed material in the preheater, forming chlorides and sulfates. This process creates an internal circulation loop, leading to their accumulation within the system [4].

Excessive buildup of these volatiles causes significant operational and quality issues, including preheater coating and blockages that impair gas flow and thermal efficiency. Furthermore, elevated alkali content in clinker can promote the deleterious alkali-silica reaction (ASR) in concrete, leading to expansion and cracking. To mitigate these problems and maintain process stability, volatiles are selectively removed via an alkali bypass system. The captured Alkali Bypass Dust (ABD) is typically collected using bag filters or electrostatic precipitators [5]. Traditionally regarded as a waste product, ABD poses considerable storage and disposal challenges. This material has different chemical and physical characteristics compared to Cement Kiln Dust (CKD) [6,7,8,9].

While both CKD and ABD are fine particulates captured from cement kiln systems, they differ fundamentally in origin, composition, and handling due to their distinct collection points and purposes. CKD originates from the main kiln exhaust gas stream, collected via baghouses or electrostatic precipitators as a byproduct of primary air pollution control, and its composition closely resembles partially calcined raw meal, rich in calcium oxide, silica, alumina, and iron oxide, with low levels of alkalis and chlorides as opposed to ABD [7,8].

Historically landfilled, ABD presents environmental risks due to its high solubility; rainwater can leach salts into soil and groundwater, potentially contaminating local ecosystems [10]. Consequently, there is growing interest in ABD as a sustainable resource for the construction industry. Recent research has explored its use as an alkaline activator, where its high alkalinity can effectively trigger the reactivity of industrial residues like ladle furnace slag and coal ash in one-part alkali-activated binders [11]. Furthermore, ABD-based binders show promise for CO_2_ capture, as controlled carbonation can enhance their strength while sequestering carbon. Other potential applications include its use as a partial replacement for natural gypsum in cement production and as a soil stabilization agent, though the latter requires careful management of its salt content [11].

A primary research focus has been the use of ABD as a partial cement replacement in mortars. However, studies have reported mixed mechanical results. Higher ABD content typically increases water demand and extends setting times due to its higher water absorption and lower reactivity compared to Portland cement. This often leads to significant reductions in compressive and flexural strength. Therefore, while high-volume replacement compromises performance, a moderate replacement level (e.g., 30%) has been deemed viable for non-structural applications like masonry and plastering [6].

To advance this potential, this study investigates the feasibility of increasing the ABD replacement percentage in cement mortars for non-structural applications (e.g., plastering, masonry), where high mechanical strength is non-critical. A moderate reduction in strength is considered acceptable in exchange for enhanced sustainability and lower cost. The methodology involves incorporating silica fume (SF) and water-reducing admixtures (WR) into mortar mixtures with variable ABD content to mitigate workability and strength losses. The findings will determine the viability of this approach for reducing the carbon footprint and cost of construction, culminating in a recommended optimal blend. Furthermore, Response Surface Methodology (RSM) is employed to develop predictive models that analyze the combined effects of ABD, WR, and SF on the mechanical performance of cement mortar influence of ABD, WR and SF on the strength performance of cement mortar.

## 2. Materials and Methods

### 2.1. Materials

In this project, the materials used are Portland cement from Riyadh cement (Riyadh, Saudi Arabia) as per ASTM C 150 [12], and the chemical composition of the cement is shown in Table 1.

ABD was collected from the bypass system installed in the kiln at the Riyadh Cement Factory (Riyadh, Saudi Arabia). The X-ray fluorescence (XRF) spectroscopy technique (Bruker-S8 TIGER series) [13] was selected to analyze the chemicals composing ABD as shown in Table 2.

The specific gravity for the ABD was determined to be 2.168 as per ASTM C188 [14]. The sieve analysis is shown in Table 3 as per ASTM C430 [15]. The standard sand from Normalise (Leucate, France) as per ASTM C C778 [16] was used to produce mortar mixtures. Silica fume (SF), conforming to ASTM C1240 [17], was used, and mid-range water reducers, as per ASTM C494 [18], were also used. They all were brought from Sica company (Riyadh, Saudi Arabia).

### 2.2. Research Aim and Experimental Approach

This study investigates the potential utilization of waste Alkali Bypass Dust (ABD), a byproduct of cement manufacturing, as a supplementary cementitious material (SCM) in mortar formulations. Preliminary trials revealed that the direct incorporation of ABD as a partial replacement for ordinary Portland cement (OPC) detrimentally impacted both fresh-state properties (notably workability) and hardened-state properties (compressive and flexural strength). To counteract these deleterious effects, a dual-modification strategy was implemented: (1) the introduction of a mid-range water-reducing admixture (WR) to ameliorate workability loss, and (2) the incorporation of silica fume (SF) at 5% by weight of total cementitious materials to mitigate strength reduction through pozzolanic activity and pore refinement.

### 2.3. Mixture Design and Proportions

A control mortar mixture was designed with a cement-to-sand ratio of 1:3 by weight. The effective water–binder ratio (w/b) was recalculated for each mix to maintain a fixed w/c ratio of 0.5, consistent with standard mortar practices. OPC was subsequently replaced by ABD at levels of 0% (control), 10%, 30%, and 50% by weight. To systematically evaluate the efficacy of the mitigation strategy, four distinct series of mixtures were prepared:

- Series A (ABD Only): Baseline mixtures containing ABD as the sole cement replacement.
- Series B (ABD + WR): Mixtures identical to Series A but with a WR admixture. The WR dosage was calibrated for each ABD replacement level to achieve a target mortar flow of approximately 100%, thereby normalizing workability and isolating its effect on subsequent properties.
- Series C (ABD + SF): Mixtures replicating Series A with the addition of a fixed 5% silica fume by weight of cementitious materials. This series was designed to assess the independent contribution of SF to strength development.
- Series D (ABD + WR + SF): Mixtures incorporating both modifications—WR (dosage adjusted to maintain ~100% flow) and 5% SF—representing the comprehensive mitigation approach.

The details of the proportions for the mortar mixture for each series are shown in Table 4.

The mortar mixtures were prepared and mixed as per ASTM C 305-20 [19] and placed in molds measuring 4 cm × 4 cm × 16 cm to produce specimens for compressive and flexure strength measurements according to ASTM C 349 [20] and ASTM C348 [21], respectively, at 3 days, 7 days, and 28 days. After demolding, the specimens were stored in a curing tank at 25°.

### 2.4. Statistical Analysis Using Response Surface Methodology (RSM)

Response Surface Methodology (RSM) was applied to develop predictive models and to examine the combined influence of ABD, WR and SF on the strength performance of cement mortar. Design Expert^®^ software (V 13.0) was used for experimental design, statistical analysis and prediction. A custom design approach was selected because the factor levels were predefined based on preliminary trials, material behavior and the requirement to maintain workable mixes. The study included three independent variables. ABD was incorporated at four levels, namely 0, 10, 30 and 50 percent by weight of cement. WR dosage ranged from 0 to 2.6 percent, and the required amount for each mix was adjusted to achieve a uniform flow value of 100 mm. SF was used at two replacement levels, 0 and 5 percent by weight of cement. These factor ranges were selected to evaluate the individual and combined effects of additives on both early and later age strength development. The measured responses consisted of the compressive and flexural strengths at 3, 7 and 28 days. These response variables were selected to represent the progressive development of mechanical properties. After analyzing the experimental data, Design Expert^®^ recommended the two-factor interaction (2FI) model as the most appropriate model type for the responses based on higher R_2_ (>0.80) and lower *p*-value (<0.05) [22]. The 2FI model allowed the identification of significant pairwise interactions among ABD, WR and SF, and facilitated the development of predictive equations for each strength parameter. The general form of the 2FI regression model used in this study is presented in Equation (1) [23].

(1) $$Y=\beta_{0}+\beta_{1}X_{1}+\beta_{2}X_{2}+\beta_{3}X_{3}+\beta_{12}X_{1}X_{2}+\beta_{13}X_{1}X_{3}+\beta_{23}X_{2}X_{3}$$

where Y = predicted response (3d, 7d, or 28d compressive or flexural strength), X_1_ = ABD, X_2_ = WR, X_3_ = SF, β_0_ = intercept (constant term), β_1_, β_2_, β_3_ = linear coefficients, β_12_, β_13_, β_23_ = coefficients representing the interactions between pairs of variables.

## 3. Results

### 3.1. Compressive and Flexure Strength Measurements for Mortar Mixtures

**Figure 1:**
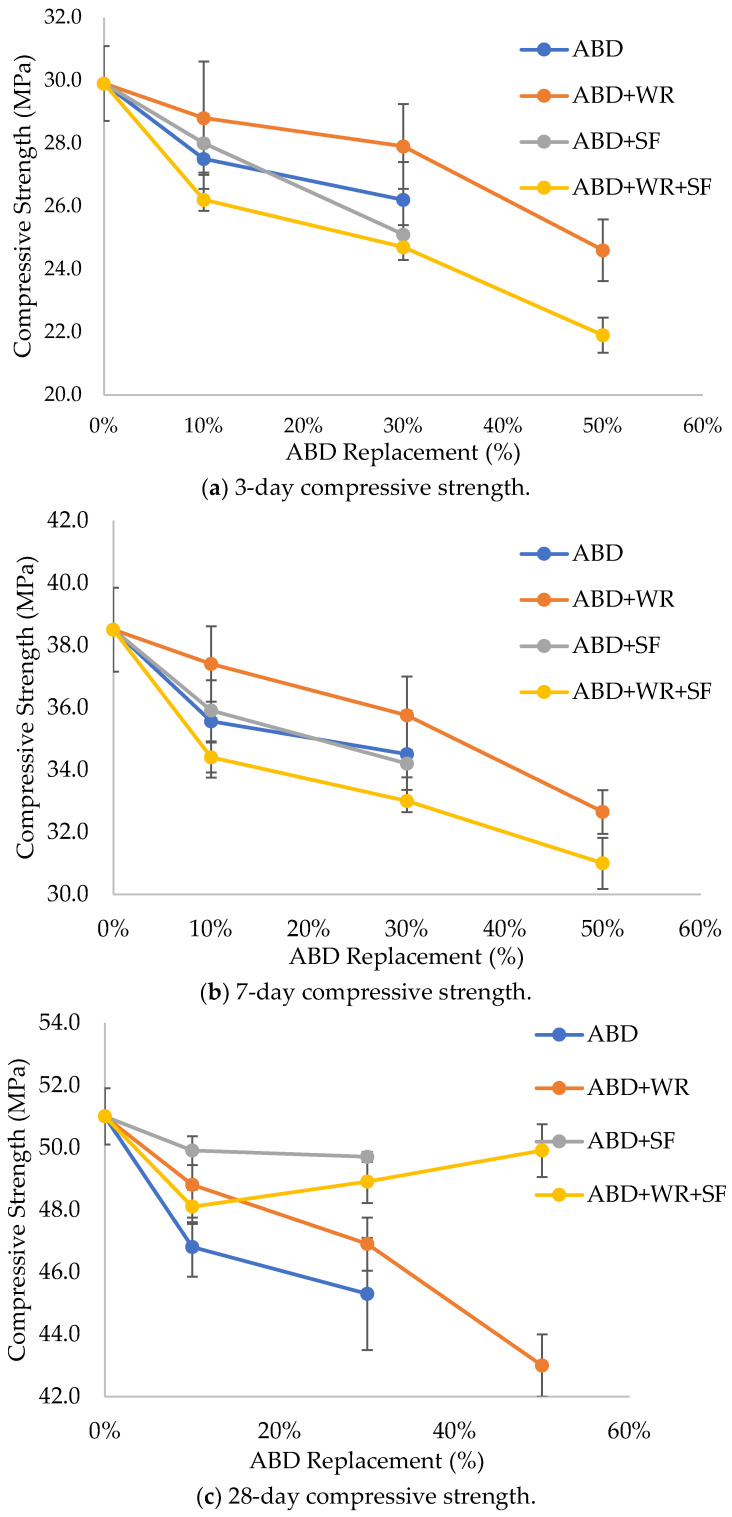

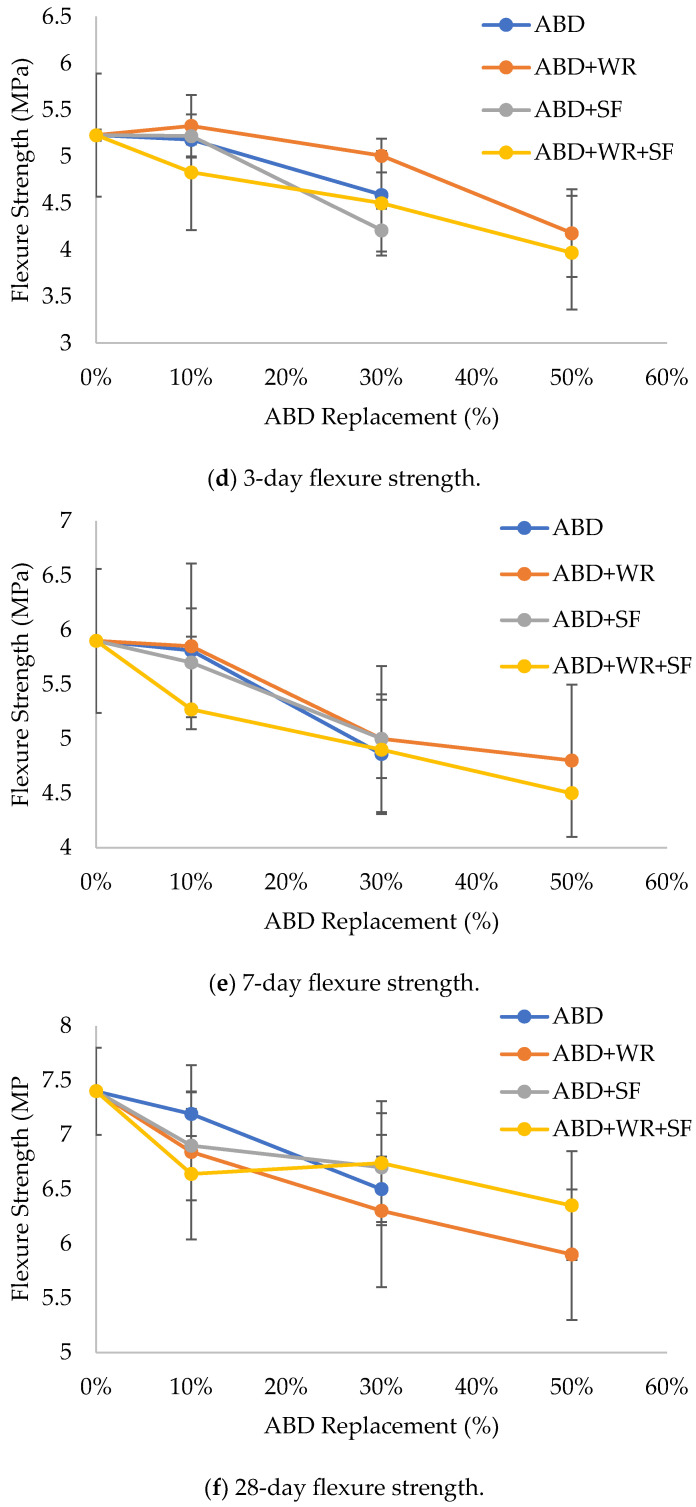
The compressive strength performances of mortar mixture series A (ABD only), series B (ABD + WR), series C (ABD + SF), and series D (ABD + WR + SF) at (**a**) 3 days, (**b**) 7 days, and (**c**) 28 days. Plus, the flexural strength performances of mortar mixture series A (ABD only), series B (ABD + WR), series C (ABD + SF), and series D (ABD + WR + SF) at (**d**) 3 days, (**e**) 7 days, and (**f**) 28 days.

The effect of incorporating WR and SF on the compressive and flexure strength of mortar mixtures containing ABD is shown in Figure 1.

The compressive strength results, presented in Figure 1a–c, demonstrate a clear trend: the incorporation of ABD as a cement replacement led to a reduction in compressive strength monotonically with increasing ABD replacement levels (0% to 50%) across all ages (3, 7, and 28 days). This is likely attributable to ABD acting as a partial cement replacement, which reduces the clinker-based binder content. Since cement is the primary source of hydration products, its reduction directly results in fewer binding phases and, consequently, lower strength.

The inclusion of a WR (Series B: ABD + WR) results in a notable improvement in early-age strength development. At 3 and 7 days, compressive strengths for Series B mixtures showed significant improvement compared to both the unmodified ABD mixtures (Series A: ABD) and those containing only SF (Series C: ABD + SF). This enhancement can be attributed to the WR’s role in dispersing the binder particles more effectively, improving the paste’s fluidity, directly enhancing the strength by reducing capillary porosity and increasing paste density. The use of WR is essential to mitigating the negative impact of high water demand associated with ABD. At 50% replacement, the ABD + WR mixtures still perform considerably better than their non-WR counterparts.

For mixtures containing only SF as an additive (Series C), the early-age (3- and 7-day) compressive strengths showed negligible change relative to the baseline (Series A), as shown in Figure 1a,b, which was contrary to expectations because SF is known for its high early reactivity. However, high levels of alkalis from ABD may, in some cases, alter the early hydration kinematics when combined with highly reactive silica, potentially causing retardation. This can be investigated in future research by measuring the hydration heat or setting time data to confirm the retardation as the specific mechanism.

However, a significant strength gain was observed at 28 days, shown in Figure 1c. This delayed effect is characteristic of pozzolanic reactions, wherein SF reacts with calcium hydroxide liberated during cement hydration to form additional calcium silicate hydrate (C-S-H) gel. The similarity in 28-day strength for mixtures with 10% and 30% ABD suggests that, at these levels, the strength-enhancing effect of the 5% SF partially compensated for the dilution effect caused by the ABD.

The complete mitigation series (Series D: ABD + WR + SF) exhibited complex behavior. At early ages (3 and 7 days), these mixtures recorded the lowest compressive strengths across all series, despite the presence of both modifying agents as seen in Figure 1a,b. This indicates a potential early-age interaction or retardation effect when both admixtures are present with high ABD content (future research). In contrast, the 28-day strength profile for Series D changed markedly. Contrary to the trend observed in other series, the 28-day compressive strength increased with higher ABD replacement, reaching its peak at the 50% replacement level, as observed in Figure 1c. This suggests a synergistic, time-dependent interaction where the SF may not only undergo its own pozzolanic reaction but also potentially activate latent hydraulic or pozzolanic properties in the ABD over time, leading to substantial late-strength development. This can be justified with the use of X-ray diffraction (XRD) and Scanning Electron Microscopy (SEM) in future research.

Meanwhile the WR allowed the ABD replacement to reach 50% without impacting the workability.

The flexural strength results, shown in Figure 1d–f, mirror the compressive strength trends but display greater sensitivity and variability. In Figure 1f, the 28-day flexural strength shows series D (ABD + WR + SF) and series C (ABD + SF) mixes performing notably well at high replacement rates (30% and 50%), sometimes even better than series A (ABD-only) and series B (ABD + WR) mixes, which is similar to the 28-day compressive results. This further supports the hypothesis that the beneficial micro-filling and pozzolanic effects of SF are long-term, particularly useful at densifying the matrix at higher replacement percentages.

Despite exhibiting reduced early-age (3- and 7-day) compressive strength, series C (with 30% cement replaced by ABD + SF) and series D (with 50% cement replaced by ABD + SF + WR) achieved 28-day strengths within 2% of the control mixture. Notably, series D incorporated a higher ABD content (50%), which enhances sustainability and reduces cost while maintaining high performance. In contrast, tensile strength demonstrated a moderate reduction relative to the control. The resulting mortars, particularly with 30% ABD replacement (series C) and 50% replacement (series D), remain suitable for non-structural building elements such as plastering and masonry. While these applications do not contribute to the primary load-bearing system, they require sufficient compressive strength to support self-weight, withstand handling, and resist incidental impacts. The strength requirements for such elements are substantially lower than for structural members. For instance, relevant ASTM standards specify compressive strength ranges of 0.5–17 MPa for masonry mortar (ASTM C270 [24]), 1–15 MPa for plaster (ASTM C926 [25]), and 13–40 MPa for masonry units (ASTM C90 [26]). The measured 28-day compressive strength of approximately 49.9 MPa for both series C and D far exceeds these thresholds.

Therefore, replacing 30–50% of cement with ABD presents a practical and sustainable alternative for non-structural mortars. This approach not only meets requisite performance standards but also promotes sustainability by valorizing an environmentally detrimental waste product. Additional benefits include reduced material costs for masonry and plastering work and a lower carbon footprint from cement production. While this study confirms short-term viability, further durability investigations are recommended to assess long-term performance. Given the minimal compressive strength reduction observed, future work should also explore the potential for using ABD-modified mixtures in structural applications.

### 3.2. Statistical Analysis Using RSM

#### 3.2.1. RSM Analysis of Compressive Strength

The response surface analysis, Table 5, showed that the compressive strength models at 3, 7, and 28 days were statistically significant. The F values of 34.63, 29.24, and 27.67 with *p* values lower than 0.0001 indicate that the experimental factors had a strong influence on strength development. The R2 values ranged between 0.8020 and 0.8352, which demonstrates that the models explained more than eighty percent of the variation in the measured strength [27]. The adjusted and predicted R2 values were also in close agreement, confirming the consistency and reliability of the fitted models [28]. In addition, the Adequate Precision values were much higher than the required minimum value of four. This shows that the models had a strong signal to noise ratio and were suitable for navigating the design space.

The results highlight the combined effect of ABD, SF, and WR on the compressive strength of the mortar. The identification of a two-factor interaction model for all curing ages suggests that the response depended strongly on the interaction among the materials rather than on individual effects alone. This outcome aligns with the expected behavior of blended cementitious systems where the presence of supplementary materials alters hydration reactions, pore structure, and matrix densification. SF enhances packing and contributes to secondary calcium silicate hydrate, while the alkaline components in ABD can influence early hydration and accelerate or modify the formation of binding compounds. The WR affects moisture availability at early age and may influence the long-term microstructure. The strong interaction among these three variables is therefore a key factor behind the observed strength variations.

Overall, the statistical performance of the models confirms that the chosen materials significantly influenced compressive strength at all curing ages. The high model fit and strong precision values demonstrate that the response surface models were robust and can be used to optimize the proportioning of ABD, SF, and WR for improved compressive performance.

**Figure 2:**
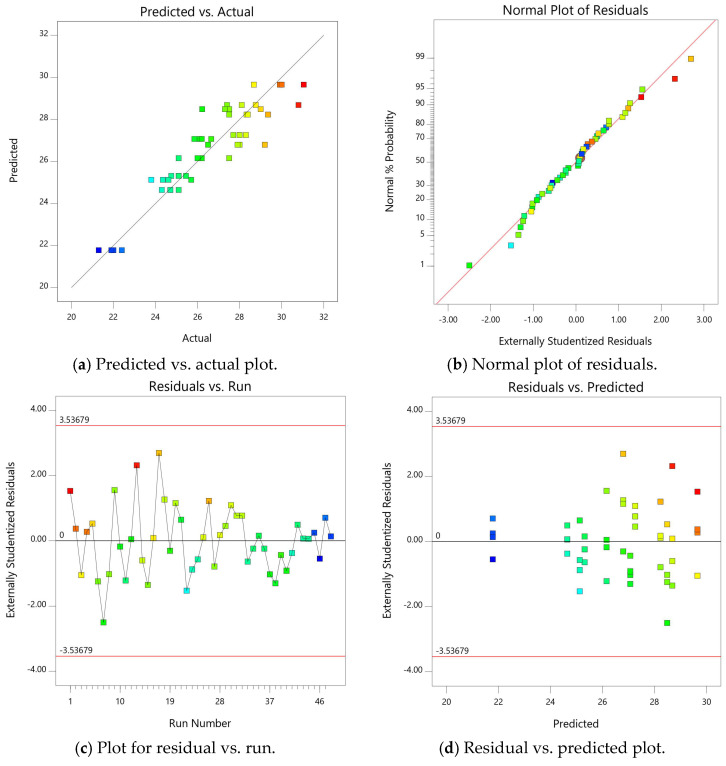
Diagnostic plots for 3d compressive strength.

The diagnostic plots shown in Figure 2 for the 3-day compressive strength indicate that the developed model captures the early-age strength behavior with good accuracy. The predicted versus actual plot (Figure 2a) shows a well aligned cluster of points along the reference line, which reflects a strong agreement between experimental and model-generated values even at this immature stage of hydration and is also depicted from higher R^2^ values (>0.80). The normal probability plot of residuals (Figure 2b) exhibits an orderly trend along the straight line, suggesting that the residuals follow an approximately normal distribution and that no major deviations or heavy tails exist. The residuals versus run plot (Figure 2c) shows a dispersed pattern without any repeating cycles, confirming that the errors are independent and not influenced by the sequence of experimental runs. The residuals versus predicted plot also (Figure 2d) shows a random and uniform distribution of points around the zero line, which demonstrates that there is no visible increase or decrease in error magnitude with respect to predicted strength. These observations collectively confirm that the model is statistically reliable for describing early-age strength development. The model significancy and relationship between actual and predicted data is also confirmed from higher R^2^ values (>0.80) as shown in Table 5.

**Figure 3:**
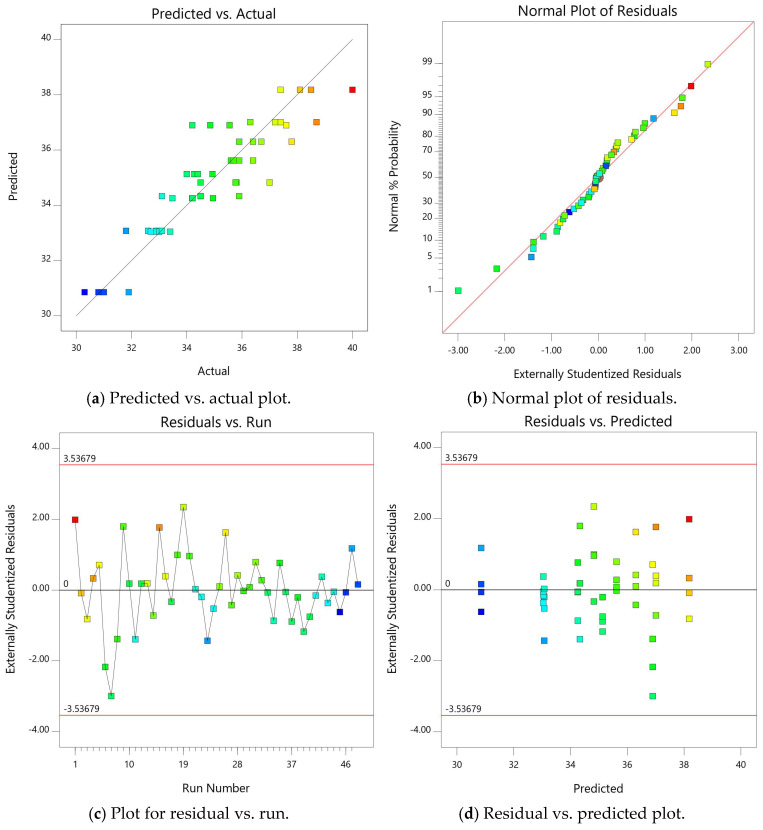
Diagnostic plots for 7d compressive strength.

The diagnostic results for the 7-day compressive strength further support the adequacy of the fitted model as presented in Figure 3a–d. The predicted versus actual plot for 7d CS reveals stronger linearity compared to the 3-day data, implying an improvement in model accuracy as the mortar gains structural maturity and hydration products stabilize. The normal probability plot shows that the majority of points lie close to the reference line, with only minor deviations at the tails, which is acceptable for RSM modeling at intermediate curing periods. The residuals versus run plot present a scattered but well balanced distribution of residuals across the run order, indicating that experimental errors remained consistent throughout the test series and were not affected by operational factors such as batching or curing sequence. The residuals versus predicted plot display a homogeneous spread of points with no evident funneling or clustering, showing that the model does not suffer from heteroscedasticity. These indicators confirm that the 7-day model performs well and provides stable predictions across the full factor range.

**Figure 4:**
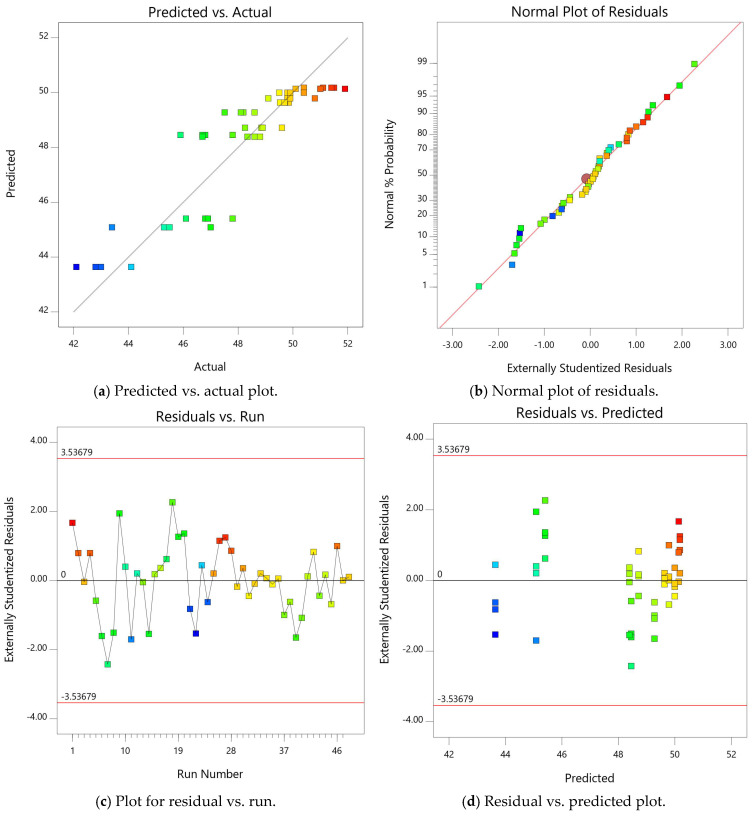
Diagnostic plots for 28d CS.

The diagnostic plots for the 28-day compressive strength show that the model performs reliably even at the fully matured stage of mortar development as depicted in Figure 4a–d. The predicted versus actual values demonstrate a tight grouping of points along the line of equality, with only a few mild deviations at higher strength levels, suggesting strong predictive capability for long-term strength. The normal probability plot reveals a smooth and consistent alignment of residuals along the straight line, which indicates that the assumption of normality is satisfactorily met at this age. The residuals versus run plot show an even distribution of residuals without any systematic drift, meaning that no time-related or process-related bias existed during the extended testing period. The residuals versus predicted plot show a balanced dispersion around the zero line, confirming that the model does not under or over predict values at any strength range. Overall, the diagnostic patterns verify that the model captures the long-term behavior of the blended mortar effectively and is suitable for prediction and optimization of the 28-day response.

#### 3.2.2. RSM Analysis for Flexural Strength

The analysis of the flexural strength responses at 3, 7, and 28 days also confirmed strong model significance as shown in Table 6. The F values of 29.61, 31.50, and 28.38, together with *p* values below 0.0001, show that the selected variables had a meaningful effect on the flexural strength of the mortar. The R values were between 0.8059 and 0.8217, which indicates that the models captured a large portion of the variability in the responses. The adjusted and predicted R values remained consistent and within the acceptable difference margin, confirming good model stability. The Adequate Precision values exceeded sixteen for all curing ages, which demonstrates that the models had a sufficiently strong signal to noise ratio.

The two-factor interaction model was recommended for all flexural strength responses, which highlights the importance of the combined effects of ABD, SF, and WR. Flexural strength is more sensitive to microstructural quality and interfacial bonding compared with compressive strength. Therefore, the interaction among the supplementary materials plays a critical role. Silica fume is known for refining pore structure and strengthening the interfacial transition zone. ABD contributes chemical components that may influence the formation of hydration products, while the water retarder affects early-age water distribution and the later formation of microcracks. The interaction among these factors therefore becomes the dominant mechanism governing flexural performance.

The strong statistical indicators confirm that flexural strength is significantly affected by the synergy between ABD, SF, and WR. The fitted models were able to predict the responses with good accuracy and can be reliably used for optimization. These results show that the combined use of the three materials not only influences compressive strength but also plays a major role in controlling the tensile and bending behavior of the mortar.

**Figure 5:**
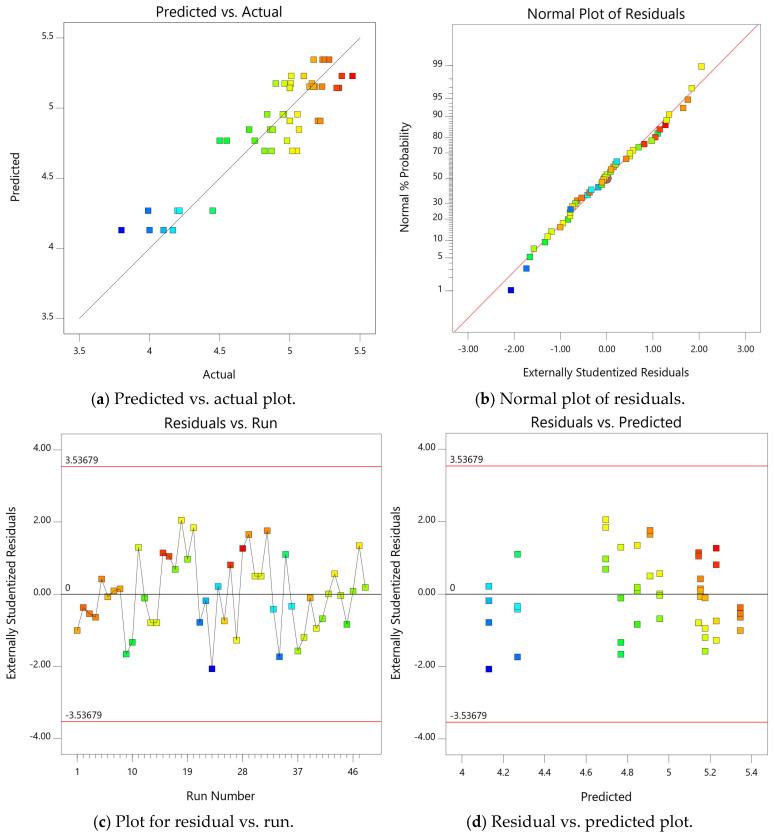
Diagnostic plots for 3d flexure strength.

The diagnostic plots for 3-day FS indicate that the RSM model captured the early-age behavior of the mortar with acceptable accuracy as illustrated in Figure 5a–d. In the predicted versus actual graph, the majority of points are closely aligned with the reference line, showing that the model fairly reproduced the measured flexural strength despite the higher variability usually seen at early ages. The normal probability plot of residuals also shows a near-linear trend with only a few mild deviations at the lower tail, suggesting that the assumption of normality was largely satisfied. The residuals versus run plot does not display any systematic waves or clusters, which means that no experimental drift or hidden time-related effects influenced the responses. Additionally, the residuals versus predicted plot confirms that the errors were randomly scattered around zero, with no expanding or funnel-like pattern. This random distribution indicates that the model did not suffer from heteroscedasticity and remained stable in predicting early-age flexural strength under the combined effects of ABD, WR and SF. The model significancy and relationship between actual and predicted data is also confirmed from higher R^2^ values (>0.80) as shown in Table 6.

**Figure 6:**
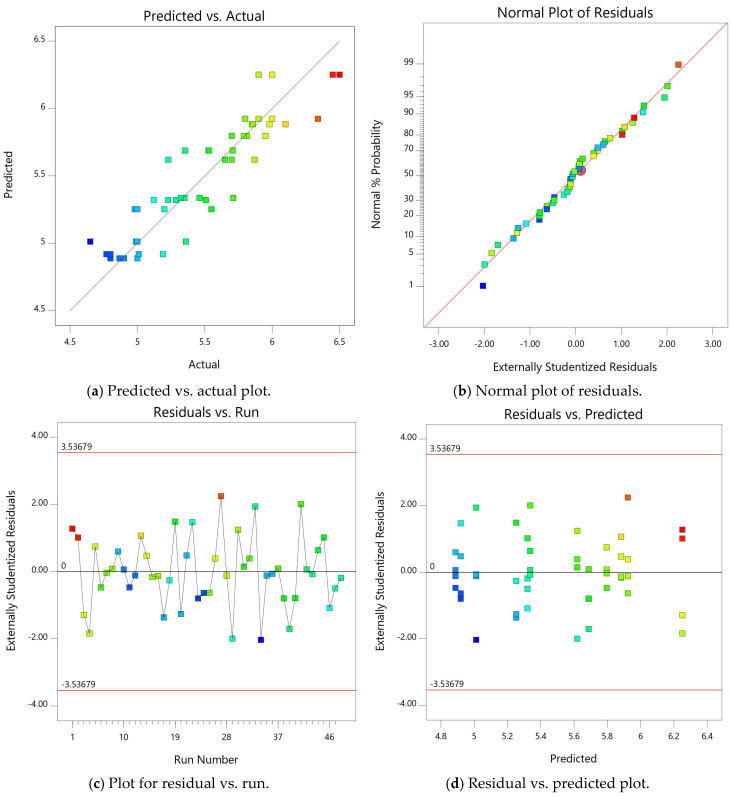
Diagnostic plots for 7-day flexure strength.

The 7-day FS diagnostic plots (Figure 6a–d) reflect a clearer improvement in model performance compared to the 3-day results. The predicted versus actual plot shows a tighter grouping along the equality line, pointing to better predictive precision at this intermediate curing age. The distribution of residuals in the normal probability plot follows the theoretical line more consistently, implying stronger compliance with normality. The residuals versus run graph demonstrates a balanced spread of positive and negative errors throughout the experimental sequence, indicating that the mixing, curing and material proportions remained consistent during the testing period. In the residuals versus predicted plot, the points remain evenly dispersed without any visible pattern, confirming that the model errors remained independent of the magnitude of predicted strength. This behavior suggests that the mortar’s microstructural development at 7 days reached a more stable phase where the influence of ABD, WR and SF interacted more predictably, enabling the RSM model to capture the flexural response with higher reliability.

**Figure 7:**
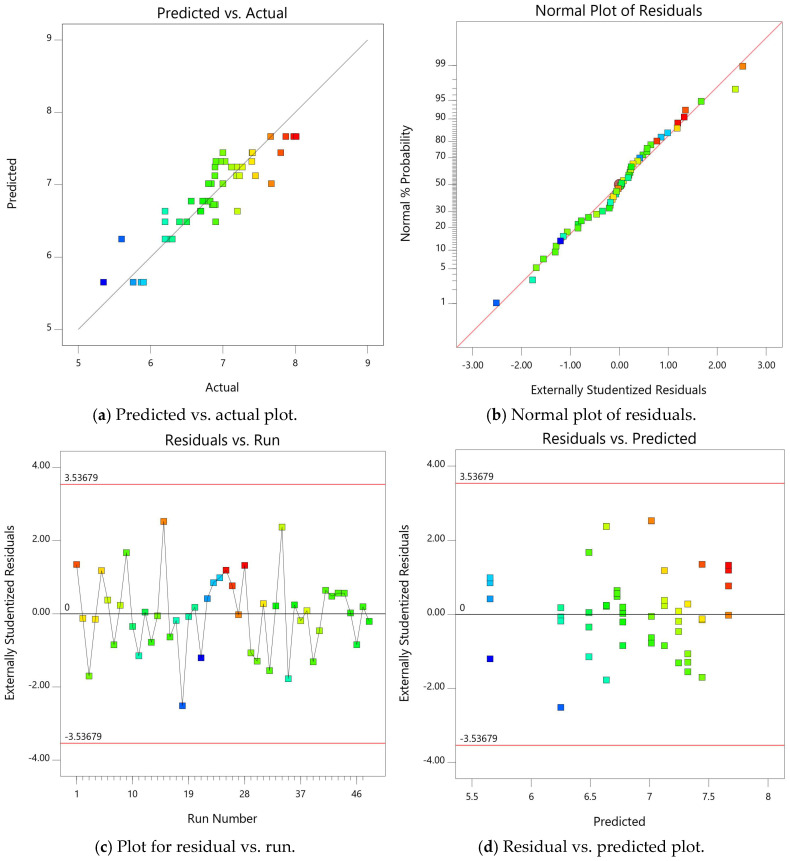
Diagnostic plots for 28d flexure strength.

The diagnostic plots for 28-day FS show the strongest statistical performance among the three ages as shown in Figure 7a–d. The predicted versus actual plot exhibits a very close alignment of points with the reference line, demonstrating that the model accurately captured long-term flexural behavior. The normal probability plot displays a nearly perfect straight-line relationship, which confirms that the residuals followed a normal distribution. This strong compliance indicates that the variability in mechanical response at a later age was well represented by the RSM model. The residuals versus run plot shows a stable and random pattern with no upward or downward drift, confirming that the experimental sequence did not influence the results. Finally, the residuals versus predicted plot shows a balanced and compact scattering of residuals around zero. The absence of any directional trend suggests that the model predictions were free from bias across the entire strength range. These results indicate that at 28 days, the combined effects of ABD, WR and SF produced a more mature and uniform matrix, allowing the model to reliably represent the flexural strength development.

#### 3.2.3. Prediction Equation of Responses

Based on a higher R^2^ and lower *p*-value the models for all responses were suggested as 2FI. The following Equations (2)–(7) are generated based on the coefficients obtained from RSM ANOVA analysis. These equations can be used to predict CS and FS at 3d, 7d and 28d curing.

(2) $${3d}_{CS}=26.65-2.82A+0.383B-1.50C-0.145AB+0.241AC-1.03BC$$

(3) $${7d}_{CS}=34.65-2.19A-0.137B-1.34C+0.267AB+0.751AC-1.15BC$$

(4) $${28d}_{CS}=47.41-0.842A-0.421B+1.09C+1.49AB+1.88AC-0.81BC$$

(5) $${3d}_{FS}=4.94-0.752A+0.292B+0.21C-0.111AB-0.159AC+0.428BC$$

(6) $${14d}_{FS}=5.67-0.788A+0.528B-0.206C+0.16AB+0.188AC-0.23BC$$

(7) $${28D}_{FS}=6.44-0.426A-0.292B+0.383C+0.402AB-0.032AC+0.303BC$$

where CS is compressive strength (MPa), FS is flexural strength (MPa), A is ABD (alkaline bypass dust), B is WR (water retarder) and C is SF (silica fume). ABD is in percentage and in the range of 0 to 50%, WR is in percentage and is in the range of 0 to 6,5 and SF is in the percentage and used in the range of 0 to 5%.

## 4. Practical Significance

This study demonstrates a viable, high-value application for ABD by successfully incorporating it as a 30–50% cement replacement in mortar. The key practical outcome is the development of sustainable mortar mixes suitable for non-structural building elements like plastering and masonry work. Specifically, the optimal mixes (Series C with 30% ABD + silica fume and Series D with 50% ABD + silica fume + water reducer) achieve 28-day compressive strengths (~49.9 MPa) that far exceed the requirements of relevant ASTM standards for such applications (0.5–40 MPa). This performance is achieved despite a moderate reduction in early strength and tensile capacity.

The results demonstrate that robust statistical models have been successfully developed to predict the compressive and flexural strength of mortar containing ABD, SF, and WR. The high model significance, precision, and diagnostic validity mean these models are reliable tools for practical application. Practically, this allows for the optimization of mortar mixes without the need for exhaustive trial-and-error testing. Engineers and mix designers can use the provided equations to input specific levels of ABD, SF, and WR and accurately forecast the resulting 3-, 7-, and 28-day strengths. This is particularly valuable for tailoring mortar properties for specific applications, such as achieving higher early strength or optimizing long-term performance, while potentially utilizing industrial byproducts like ABD.

Furthermore, the identification of a two-factor interaction (2FI) model as the best fit for all responses is of major practical importance. It reveals that the synergistic interaction between the materials, not just their individual amounts, is the dominant factor controlling strength development. This means that changing one component (e.g., SF content) will have a different effect depending on the levels of the other two (ABD and WR). In practice, this underscores the need for a holistic approach to mix design, where the combined formulation is carefully balanced. For instance, the model can help identify combinations where ABD and SF interact to maximize matrix densification or where the WR optimally manages water availability to enhance microstructural development without harming early strength.

The practical benefits of this application are the utilization of this industrial waste, which reduces landfill use and lowers the carbon footprint of construction by cutting cement demand, the direct cost-saving opportunity for masonry and plastering projects through reduced cement consumption, and the development of a practical mix design solution, showing that a combined admixture system (WR and SF) is essential to mitigate ABD’s high water demand and unlock its long-term, strength-enhancing pozzolanic potential at high replacement levels. While immediate use is recommended for non-structural purposes, the minimal strength loss observed also suggests promising potential for exploring these mixes in structural applications, warranting further durability and long-term performance studies.

## 5. Conclusions

In conclusion, this study demonstrates that using ABD as a partial cement replacement significantly alters mortar’s mechanical properties, with outcomes governed by a complex interplay between replacement level, chemical admixtures, and curing age. While the primary dilution effect of ABD leads to a monotonic decline in compressive strength, this trend can be effectively reversed through strategic combinations, such as incorporating water reducers (WRs) to counteract high water demand and silica fume (SF) to provide delayed pozzolanic activity and synergistic strength gain. Notably, mixtures with 30% ABD and SF, or 50% ABD with WR and SF, achieved 28-day compressive strengths within 2% of the control, exceeding ASTM requirements for non-structural applications like masonry mortar and plaster. This confirms that replacing 30–50% of cement with ABD in optimized blends offers a viable, high-performance, and sustainable alternative that lowers the carbon footprint while valorizing industrial waste. The Response Surface Methodology (RSM) effectively modeled these interactions, producing statistically significant predictive equations for compressive and flexural strength across curing ages, with high R^2^ values confirming that strength development is governed more by synergistic interactions than by individual factors. These models provide a practical tool for designing sustainable mortar mixes with precise control over mechanical properties. Future work should focus on long-term durability assessment and exploring these mixtures for potential structural applications.

## Figures and Tables

**Table 1:** Chemical compositions of cement [6].

Chemical Analysis	Cement	Standard Method	Specifications SASO-GSO 1914/2009
Loss on Ignition%	2.4		3.0% Max
Insoluble Residue%	0.8	ASTMC114	1.5% Max
SiO_2_	20.25		-
Al_2_O_3_%	5.06		-
FeO_3_%	4.28		-
CaO%	63.66		-
MgO%	0.74		5.0% Max
SO%	2.74		3.0% Max (C3A ≤ 8%)
Chlorides%	0.02		3.5% Max (C3A ≥ 8%)
LSF	94.33		
C3A	6.17	ASTMC150	
Total Alkalis Equivalent	0.45		<0.60 for Low Alkali

**Table 2:** Chemical compositions of ABD [6].

Chemical Analysis	SiO_2_	Al_2_O_3_	FeO_3_	CaO	MgO	Na_2_O	K_2_O	SO_3_
	14.28	5.13	0.237	44.32	0	0.11	0.12	0.07
	Cl	LOI	Moist	QV *	LSF *	SM *	AM *	Eq AlC *
	0.053	1.06	-	0.38	95.95	2.66	21.65	0.19

* QV: quotient of volatiles, LSF: lime saturation factor, SM: silica modulus, AM: alumina modulus, Eq AlC: equivalent alkali as chloride.

**Table 3:** Sieve analysis of ABD [6].

Sieve Size	45 µm	65 µm	90 µm
Average value	8.8	3.2	16.6

**Table 4:** Mixture proportions for mortar.

	Materials (Grams)	ABD 0%	ABD 10%	ABD 30%	ABD 50%
Series A	Cement	450	405	315	225
	ABD	0.0	45	135	225
	Water	225	225	225	225
	Standard Sand	1350	1350	1350	1350
Series B	Cement	450	405	315	225
	ABD	0.0	45	135	225
	Water	225	225	225	225
	Standard Sand	1350	1350	1350	1350
	WR	0	0.75	2.70	6
Series C	Cement	450	385	300	214
	ABD	0.0	42	128	213
	Water	225	225	225	225
	Standard Sand	1350	1350	1350	1350
	SF	0	22	22	22
Series D	Cement	450	385	300	214
	ABD	0.0	42	128	213
	Water	225	225	225	225
	Standard Sand	1350	1350	1350	1350
	WR	0	1.1	3.2	6.5
	SF	0	22	22	22

**Table 5:** Fit statistics and ANOVA for compressive strengths (CSs).

Model Term	Value		
	3d CS	7d CS	28d CS
Model sum of squares	206.49	182.26	221.29
Model mean squares	34.42	30.38	36.88
Model F-value	34.63	29.24	27.67
Model *p*-value	<0.0001	<0.0001	<0.0001
R^2^	0.8352	0.8106	0.8020
Adjusted R^2^	0.8111	0.7828	0.7730
Predicted R^2^	0.7891	0.7544	0.7393
Adeq Precision	20.6720	18.8175	14.8286
Std. Dev.	0.9970	1.02	1.15
Mean	26.60	34.96	48.23
Suggested Model	2FI	2FI	2FI

**Table 6:** Fit statistics and ANOVA for flexural strength (FS).

Model Term	Value		
	3d FS	7d FS	28d FS
Model sum of squares	6.24	8.39	13.96
Model mean squares	1.04	1.40	2.33
Model F-value	29.61	31.50	28.38
Model *p*-value	<0.0001	<0.0001	<0.0001
R^2^	0.8125	0.8217	0.8059
Adjusted R^2^	0.7850	0.7956	0.7775
Predicted R^2^	0.7422	0.7542	0.7349
Adeq Precision	16.9810	16.9437	18.4113
Std. Dev.	0.1874	0.2107	0.2863
Mean	4.88	5.49	6.86
Suggested Model	2FI	2FI	2FI

## Data Availability

The original contributions presented in this study are included in the article. Further inquiries can be directed to the corresponding author.

## Abbreviations

The following abbreviations are used in this manuscript:

ABD	Alkali Bypass Dust
WR	Water Reducer
SF	Silica Fume
CS	Compressive Strength
FS	Flexure Strength
